# Novel Intranasal Drug Delivery: Geraniol Charged Polymeric Mixed Micelles for Targeting Cerebral Insult as a Result of Ischaemia/Reperfusion

**DOI:** 10.3390/pharmaceutics12010076

**Published:** 2020-01-17

**Authors:** Sara M. Soliman, Nermin M. Sheta, Bassant M. M. Ibrahim, Mohammad M. El-Shawwa, Shady M. Abd El-Halim

**Affiliations:** 1 Department of Pharmaceutics and Industrial Pharmacy, Faculty of Pharmacy, 6th of October University, Central Axis, Sixth of October City, Giza 12585, Egypt; sara.soliman@o6u.edu.eg (S.M.S.); nerminsheta@o6u.edu.eg (N.M.S.); 2 Department of Pharmacology, Medical Research Division, National Research Centre, Dokki, Giza 12622, Egypt; bmmih1974@gmail.com; 3 Department of Physiology, Faculty of Medicine for Girls, Al-Azhar University, Cairo 11651, Egypt; dr_shawwa@yahoo.com

**Keywords:** ischaemia/reperfusion, geraniol, polymeric mixed micelles, behaviour, analgesic, anti-inflammatory activity

## Abstract

Brain damage caused by cerebral ischaemia/reperfusion (I/R) can lead to handicapping. So, the present study aims to evaluate the prophylactic and therapeutic effects of geraniol in the form of intranasal polymeric mixed micelle (PMM) on the central nervous system in cerebral ischaemia/reperfusion (I/R) injury. A 3^2^ factorial design was used to prepare and optimize geraniol PMM to investigate polymer and stabilizer different concentrations on particle size (PS) and percent entrapment efficiency (%EE). F3 possessing the highest desirability value (0.96), with a PS value of 32.46  ±  0.64 nm, EE of 97.85  ±  1.90%, and release efficiency of 59.66  ±  0.64%, was selected for further pharmacological and histopathological studies. In the prophylactic study, animals were classified into a sham-operated group, a positive control group for which I/R was done without treatment, and treated groups that received vehicle (plain micelles), geraniol oil, and geraniol micelles intranasally before and after I/R. In the therapeutic study, treated rats received geraniol oil and micelles after I/R. Evaluation of the effect of geraniol on behavior was done by activity cage and rotarod and the analgesic effect tested by hot plate. Anti-inflammatory activity was assessed by measuring interleukin β6, cyclooxygenase-2, hydrogen peroxide, and inducible nitric oxide synthase. Histopathogical examination of cerebral cortices was also done to confirm the results of a biochemical assay. Geraniol nanostructured polymeric mixed micelles showed an enhanced neuro-protective effect compared to geraniol oil, confirming that PMM via intranasal route could be an efficient approach for delivering geraniol directly to the brain through crossing the blood–brain barrier.

## 1. Introduction

Brain damage caused by cerebral ischaemia is an extremely serious disease due to subsequent occurrence of life-lasting disability for adults [1]. Ischaemia/reperfusion (I/R) is restoring blood flow to an organ following the incidence of ischaemia; it can be achieved by mechanical recanalization [2]. Despite the use of medical treatment for thrombolysis, as by using recombinant tissue plasminogen activator (rtPA), yet reperfusion can be put into consideration for protection against tissue damage caused by ischaemia [3]. However, there are debates about this procedure due to the risk of more serious damage that can result from haemorrhage or oedema caused by reperfusion after ischaemia [4], which is revealed as an “ischaemia/reperfusion injury” (I/RI).

I/RI of cerebral tissue is associated with the production of free radicals which in turn leads to oxidative stress and inflammation accompanied by leukocyte infiltration, destruction of the blood–brain barrier, and\or platelet activation that subsequently leads to sensory and motor disorders [1]. Oxidative stress results as an effect of oxidative phosphorylation that occurs in an attempt to restore nutrient supply and oxygenation to ischaemic cerebellum. Oxidative phosphorylation results in the production of reactive oxygen species (ROS) by the mitochondria; eventually, ROS production associated with reperfusion injury is considered as the “necessary evil” [5]; damage to cerebral tissue after I/R is referred to as “cerebral reperfusion injury” [6].

Geraniol (3, 7-dimethylocta-trans-2, 6-dien-1-ol) is a natural acyclic monoterpene alcohol with the chemical formula C_10_H_18_O. It is found in aromatic herb oils as it is the main component of rose oil and palmarosa [7]. It possesses a rose-like odor and appears as a clear to pale-yellow oil, which is soluble in most organic solvents while insoluble in water. It is also used as a flavoring agent, where it is considered and generally recognized as safe by the FDA [8]. Geraniol exhibits antioxidant [9], anti-inflammatory [10], antimicrobial, and anti-apoptotic activities [11]. Furthermore, it exerts potent antitumor activity against different types of malignancies [12].

Geraniol is readily absorbed after oral administration through the lumen of the gastrointestinal tract into the portal vein to the liver where being subjected to the first-pass metabolism results in a spacious diminishing of its oral bioavailability [13,14]. Besides the poor bioavailability, its low aqueous solubility and short half-life result in frequent administration to achieve its therapeutic activity.

Inclusion of hydrophobic drugs into nanocarrier systems such as polymeric single micelles (PM) has emerged as one of the most promising approaches for increasing their solubility, and thus, enhancing their bioavailability [15,16]. The disadvantages of mono micellar systems include low drug loading, large particle size, and limited stability [17].

One of the most commonly employed polymers to prepare PM are pluronics [18]. Pluronic-based nanocarriers are safe and undergo less opsonization than other nanocarriers since they are sterically stabilized, preventing their subsequent recognition and uptake by macrophages of the reticuloendothelial system (RES). As a consequence, PM of pluronics may have a reasonably longer half-life in circulation and may deliver the payload to desired sites of action more efficiently [19].

There are numerous reports published in literature dealing with the solubilization of hydrophobic drugs in the pluronic micelles [20,21]. Published works concerned with the use of single pluronics reported that the high critical micelle concentration (CMC) values of these polymers may be disadvantageous as the loaded micelles undergo significant dilution on administration, resulting in low stability of micelles. It has been well established that, compared to single surfactant solutions, the mixing of different type of surfactant solutions exhibit synergism in terms of better surface activities and lower CMC values. In addition, mixed micelles offer significant advantages of higher thermodynamic and kinetic stability as well as higher drug loading capacities [21,22].

Intranasal administration offers a practical, non-invasive, and rapid route of drug delivery systems. It offers the advantages of being administered simply, cost-effectively, and conveniently with improving the drug bioavailability due to bypassing the effect of the first-pass metabolism in the liver. Besides, bypassing first-pass hepatic metabolism and circumventing the brain barriers, provides a unique feature and better option for targeting drugs to the brain, as reported by Kumar et al. [23]. Ugwoke et al. stated that during designing drug delivery systems for intranasal administration, some formulation factors should be considered: “the formulation should be designed so as to provide rapid transport of the drug across nasal mucosa and a longer residence time in the nasal cavity to overcome the nasal mucociliary clearance” [24].

Therefore, the intranasal administration of mixed micelles is considered as an approach to target our drug candidate, geraniol, directly to the nose-to-brain transport pathway. The present study is conducted to evaluate the prophylactic and therapeutic effects of a novel drug delivery system targeting the central nervous system by utilizing a natural product “geraniol” to test its effect on brain damage in a model of cerebral ischaemia/reperfusion injury. Geraniol will be introduced intra-nasally and this will be the first time it is incorporated in the form of polymeric mixed micelles in a trial to achieve long time circulating intranasal nanocarriers of geraniol by enhancing its solubility and bioavailability, and thus, acquire the most available blood–brain barrier crossing effects.

## 2. Materials and Methods

### 2.1. Materials

#### 2.1.1. Chemicals

Geraniol, Pluronic^®^ F127, (Acetonitrile, methanol, and water HPLC grade), thiopental, diethylether and formaldehyde (Sigma-Aldrich, St. Louis, MO, USA); Cremophor^®^ EL (BASF, Ludwigshafen, Germany); Tween 80, hydrochloric acid, potassium dihydrogen phosphate, disodium hydrogen phosphate, and sodium chloride (Merck-Schuchardt, Hohenbrunn, Germany); synthetic cellulose nitrate membrane 0.22 µm Tuffyrn membrane filter (Sartorius Stedim, Göttingen, Germany).

#### 2.1.2. Kits

For determination of interleukin β6 (ILβ6), cyclooxygenase-2 (COX-2), hydrogen peroxide (H_2_O_2_), using enzyme-linked immune sorbent assay (ELISA) kits purchased from the MY Biosource company distributor in Egypt (Science and Technology Center), and inducible nitric oxide synthase (iNOS), using ELISA kits purchased from the Cusabio company distributor in Egypt, Indomedix.

#### 2.1.3. Animals

Male Wister albino rats weighing 250–280 g were used throughout the experiments. The animals were obtained from the animal house colony of the National research center, Dokki, Giza, Egypt. The animals were housed in standard metal cages in an air-conditioned room at 22 ± 3 °C, 55 ± 5% humidity, and provided with standard laboratory diet and water ad libitum. Experiments were performed between 9:00 and 15:00. All experimental procedures were conducted in accordance with the guide for care and use of laboratory animals and the animal procedures were performed in accordance with the Ethics Committee of the National Research Centre with approval certificate registration number 18124, and followed the recommendations of the National Institutes of Health Guide for Care and Use of Laboratory animals.

#### 2.1.4. Apparatus for Pharmacological Study

Grid Floor Activity cage (Model No. 7430; Ugo Basile, Varese, Italy) and Rotarod (Model No. 7750; Ugo Basile, Varese, Italy) were used for behavior stress tests to examine psychological and locomotor activities, respectively. Hot plate (Model No. 7280; Ugo Basile, Varese, Italy) was used for thermal tests for analgesic effect evaluation. A light microscope was used for examining tissue for histopathology.

### 2.2. Optimization of the Preparation Technology

In order to optimize the mixed micelle compositions, some preparative independent variables, namely, concentration of polymer and stabilizer at three levels each were involved to study their impact on the entrapment efficiency and particle size using Design Expert^®^ software (Version 7, Stat-Ease Inc., Minneapolis, MN, USA). For that, nine formulae were fabricated and are summarized in Table 1.

### 2.3. Preparation of Geraniol-Charged Mixed Micelles

Geraniol-loaded Pluronic^®^ F127/Cremophor El mixed micelles (MM) were prepared using a film hydration method [25,26]. In a round-bottom flask, geraniol (3% *w/v*), Pluronic^®^ F127 (3%, 6%, and 9% *w/v*) and Cremophor El (0%, 2%, and 4% *w/v*) were dissolved in 15 mL methanol and then the solvent was removed using rotary evaporator (Rotavapor, Heidolph, Schwabach, Germany) under vacuum at 30 °C for about 10 min. The formed thin film was hydrated with 10 mL of deionized water and sonicated (Julabo, Seelbach, Germany) at 37 °C for 15 min. Finally, geraniol MM was filtered through a 0.22-μm nylon syringe filter to remove the un-entrapped drug and stored at room temperature for 48 h in well-sealed glass vials before further characterization [27].

### 2.4. Characterization of Geraniol-Loaded Mixed Micelles

#### 2.4.1. Particle Size, Polydispersity Index, and Zeta Potential Determination

Particle size analysis and polydispersity index (PDI) were performed by Malvern zetasizer (Nano ZS-90, Malvern Instruments, Worcestershire, UK) for the different prepared geraniol mixed micelles. Exactly 0.1 mL of the prepared micelle was diluted with 10 mL of deionized water. The same procedure is carried out for zeta potential (ζ) measurement. The zeta potential was calculated on the basis of the electrophoretic mobility and surface charge of formulated PMMs [28]. All samples were performed in triplicate.

#### 2.4.2. Determination of Drug Loading (DL) and Encapsulation Efficiency (EE)

Both DL and EE were determined using HPLC (LC-20AD liquid chromatograph, Shimadzu, Japan) consisting of two LC-10ADvp pumps, a DGU-20A continuous degassing unit equipped with an SIL-20A autosampler and an SPD-20A UV–vis detector. Chromatographic separations were achieved by a Pronto SIL^®^ RP-C18 (150 × 4.6 mm, 5 μm) column (type SC-150, Bischoff Chromatography, Berlin, Germany).

Data acquisition and processing were performed on a personal computer using LC solution Software, version 1.25 SP4 (Shimadzu, Kyoto, Japan). The detector was set at 210 nm and the temperature was maintained at 25 °C. The mobile phase consists of an isocratic mixture of acetonitrile and deionized water (50/50, *v/v*) at a flow rate of 1 mL/min [29]. The retention time obtained was 6.9 min for geraniol.

Briefly, a known amount of MM was dissolved in methanol to quantify the geraniol concentration. The *DL* and *EE* were calculated by the following equations:

(1) DL(%)=(Weight of drug in MM/Weight of the feeding polymer and drug)×100

(2) EE(%)=(Weight of drug in MM/Weight of the feeding drug)×100

#### 2.4.3. In-Vitro Drug Release Study

The release profile of geraniol from optimized mixed micelles formulae was studied using the dialysis method [30,31]. In brief, samples were added into previously soaked dialysis bags (HIMEDIA, molecular weight cut off 12,000–14,000 KD). The dialysis bags were tied and placed into 500 mL phosphate buffer saline, pH 6.8 containing 1% Tween 80 (*w/v*).

The drug release was carried out at 100 rpm and 37 ± 1 °C for 24 h. At defined time intervals, samples of 1 mL were withdrawn and replenished with an equal volume of fresh release medium. The drug concentration at each sampling point was measured using the previously mentioned HPLC method at 210 nm. Percentage release efficiency (%RE) was estimated to compare the release of geraniol from the different selected formulae by calculating the area under the release curve values (AUC) at 24 h using the trapezoidal rule method.

#### 2.4.4. Fourier Transform Infrared Spectroscopy (FTIR)

ATR-FTIR spectroscopy is a well established tactic in literature that has several applications for qualitative and quantitative analysis of different samples and structurally related compounds [32,33]. The structural transformations of geraniol oil, plain formula (F3, the same components without adding geraniol oil), and the optimal polymeric mixed micelles formula (F3) were investigated by FTIR spectroscopy using an IRAFFINITY-1 FTIR device (Shimadzu, Kyoto, Japan). Samples were placed over the ATR ZnSe crystal 4 mm thick, 10 mm wide and 80 mm long. Scans were performed in a range from 4500 to 450 cm^−1^ with a resolution of 4 cm^−1^ and the average number of scans for each sample was 45 times.

#### 2.4.5. Transmission Electron Microscope (TEM)

A drop of the optimal formulation was placed onto 300-mesh copper grids coated with carbon without staining. Then, the copper grid was dried for about 30 min and subjected to TEM examination (Jeol, JXA-840A, Akishima, Japan) [34].

### 2.5. Pharmacological Study

#### 2.5.1. Determination of Tolerable Non-Irritant Dose

“The National Pesticide Information Center” reported 23 incidents between 1 April 1996 and 30 March 2016 of dermal or respiratory side effects as irritation and discoloration of skin when geraniol was involved as an active ingredient [35]. Therefore estimation of its effect when instilled intranasally in rats was done prior to starting the efficacy study in order to choose the suitable non-irritant volume that could be instilled into the animal’s nostrils. However, it is noteworthy that it did not produce any dermal irritation in guinea pigs [36].

##### Determination of Geraniol Oil Tolerable Dose

Ten rats were classified equally into negative control that received 0.2 mL saline intranasally (IN) and a treated group that received 0.2 mL geraniol oil IN, which was the maximum volume of the viscid oil that could be instilled into the rats’ nostrils. The rats were observed over the following 24 h for changes in behavior, bowel habits, or death. Then the rats were observed over the following 15 days for changes in behavior, weight, or bowel habits.

The dose was found to be an irritant to the rats’ nostrils and produced severe congestion and led to severe irritation of animals, but no other critical changes or deaths occurred. Then the fifth and tenth dose (0.04 and 0.02 mL) were tested in two groups, each of five rats, compared to the negative control group of five rats. The 0.04 mL dose produced minimal congestion, while the 0.02 mL dose did not produce any congestion of nostrils or change in behavior so it was selected as a reference for comparison with the effects of the novel DDS of geraniol in the efficacy study.

##### Determination of Geraniol Mixed Micelles Tolerable Dose

Ten rats were classified equally into negative control that received 0.5 mL saline IN; this was the maximum volume of the fluid that could be instilled into rats’ nostrils. The treated group received 0.5 mL geraniol micelle IN. The rats were observed over the following 24 h for changes in behavior, bowel habits, or death. No deaths occurred, and no irritation or congestions of nostrils happened. Then the rats were observed over the following 15 days for changes in behavior, weight, or bowel habits. Therefore, this dose and its half dose (0.5 and 0.25 mL) were selected for the efficacy study as they did not produce any irritation or congestion.

#### 2.5.2. Efficacy Study

The study was designed as a prophylactic and therapeutic study.

Prophylactic study: Forty-eight rats were enrolled in the study and equally divided into six groups: the first was sham-operated, for which a cervical incision was done and sutured without the induction of cerebral ischaemia/reperfusion injury; the second was a positive control for which there was cerebral injury by ischaemia/reperfusion (I/R) without receiving geraniol oil or drug-loaded micelles or vehicle (plain micelles); the third to sixth were treated groups that received vehicle (plain micelles 0.5 mL), geraniol oil (0.02 mL), geraniol micelle (0.25 mL), and geraniol micelle (0.5 mL). All tested agents were instilled in rats’ nostrils of every group once every 24 h for three successive days prior to the induction of I/R, then once every 24 h for another three successive days after induction of I/R. Rats in all treated groups received six doses of treatment once every 24 h.

Therapeutic study: The dose of geraniol micelle tested in the therapeutic study was selected based on the results of the best dose effect obtained from the prophylactic study. Thirty-two rats were enrolled in the study and equally divided into four groups: the first was sham-operated; the second was a positive control for which cerebral I/R was done and did not receive any treatment; the third and fourth were treated groups that received geraniol oil (0.02 mL) and geraniol micelle (0.5 mL), respectively. All tested agents were instilled in rats’ nostrils (IN) of every group one hour after induction of I/R. Rats in all treated groups received a single dose of treatment after the induction of I/R.

#### 2.5.3. Cerebral Ischaemia/Reperfusion (I/R) Injury Induction

The animals were kept fasting for 12 h before surgery and then anesthetized with thiopental (50 mg/kg; i.p.) [37]. A longitudinal cervical incision (2 cm) was made lateral to the midline, and the left common carotid artery (CCA) was carefully dissected. Ischaemia was induced by placing a non-traumatic micro-vascular clip on the left CCA just prior to its bifurcation [38]. During ischaemia, rats were monitored for body temperature constant at 36.5 ± 0.5 °C using a heating pad and respiration pattern. The vascular occlusion was maintained for 30 min, and then the clips were removed to resume blood flow to the ischaemic region [39]. Finally, the incisions were sutured, the animal was allowed to recover from anesthesia, and returned to a warm cage for recuperation during the reperfusion period for 24 h.

#### 2.5.4. Behavior Stress Tests

In the prophylactic study, they were done at zero time (base-line pre-ischaemic/reperfusion injury), then at 72 h (third day) after instillation of treatment and before I/R injury. While after the induction of I/R injury, they were done 24 h later and finally at 72 h after I/R injury with the continuation of instillation of treatment. In the therapeutic study, they were done before I/R at zero time but were not done after I/R or treatment as there were paralysis and deformities of the animals’ limbs.

##### Evaluation of the Psychological State of Rats Using Grid Floor Activity Cage Test

Levels of activity were measured by detecting rat movements using a grid floor activity cage (Model No. 7430; Ugo Basile, Varese, Italy), according to the method described by Pavic et al. [40].

The number of rat movements was detected by automatically recording the number of oscillations that take place as a result of horizontal animal movements across the grid floor during a five-minute test session. Oscillation information was processed in the activity cage software to provide an index of horizontal movements. Rats were acclimatized to the testing room for one hour before starting the test.

##### Evaluation of Motor Coordination Using the Rotarod Test

Motor coordination in this study was assessed using an accelerating rotarod (Model No. 7750; Ugo Basile, Varese, Italy), according to the procedure described by Vijitruth et al. [41].

Rats were acclimatized for one hour to the test room before doing the test. They were first put on the stationary rod. After getting accustomed to the rotarod, they were given a daily three training sessions separated by ten minutes on three successive days. All rats in the present study were pre-trained on the rotarod apparatus at a fixed speed of four rotations per minute (rpm) in order to reach a stable performance before starting oral treatment with the tested drugs. On the fourth day, the rats were placed on the testing rod and the speed of the rotarod was started at 4 rpm and then increased gradually to reach 40 rpm over 300 s, the average time in seconds spent on the rod for each rat was detected individually; the time until the rat fell was a measure of balance of each rat.

#### 2.5.5. Thermal Test Done by Using a Hot Plate for Evaluation of the Analgesic Effect

It was performed using a modified thermal test according to Eddy and Leimbach [42], by using an electronically controlled hot-plate (Model No 7280; Ugo Basile, Varese, Italy) adjusted at 52 ± 0.1 °C, and the cut-off time was 60 s. The time elapsed until either paw licking or jumping occurred was recorded. It was done in the prophylactic study at zero time (base-line pre-ischaemic/reperfusion injury), then 72 h after the instillation of treatment and before I/R injury, while after the induction of I/R injury, it was done 24 h later and finally 72 h after I/R injury with the continuation of instillation of treatment. In the therapeutic study, it was done before I/R at zero time but was not done after I/R or treatment as the animals were paralyzed.

#### 2.5.6. Biochemical Parameters

In the prophylactic study, animals were kept fasting for 24 h at the end of the experiment (72 h after I/R), then blood was withdrawn from the retro-orbital plexus of rats under anaesthesia with diethyl ether and centrifuged at 2500 rpm for 15 min [43]. The serum was separated and collected for the determination of ILβ6 and COX-2. Brains were dissected and homogenized for detection H_2_O_2_ and iNOS.

The assays are a solid-phase sandwich-type system that utilizes specific anti-rat ILβ6, COX-2, H_2_O_2_, iNOS antibody coated onto the wells of microtitre plates. The samples and standards were pipetted in triplicate into appropriate microtitre wells, and the assays were performed according to the manufacturer’s instructions.

In the therapeutic study, blood was withdrawn and brains were dissected under anaesthesia two hours after treatment, due to the high mortality rate.

#### 2.5.7. Brain Tissue Sampling and Preparation

At the end of the experimental period, the animals were kept fasting for 12 h and sacrificed by decapitation, then the whole brain of each animal was rapidly dissected, thoroughly washed with isotonic saline, dried and weighed. Then, each brain was sagittally divided into two portions. The first portion of each brain was homogenized immediately to give 10% (*w/v*) homogenate in ice-cold medium containing phosphate buffer (pH 7.4), then centrifuged at 4000 rpm for 15 min at 4 °C [44]. The supernatant (10%) was separated for biochemical analysis (H_2_O_2_ and iNOS).

The second portion was fixed in a formaline buffer (10%) for 24 h. Washing was done in tap water, then serial dilutions of alcohol (methyl, ethyl and absolute ethyl) were used for dehydration. Specimens were cleared in xylene and embedded in paraffin at 56 °C in a hot air oven for 24 h. Paraffin bees wax tissue blocks were prepared for sectioning at 4 microns by microtome. The obtained tissue sections were collected on glass slides, deparaffinized, and stained by hematoxylin and eosin stains [45], for histopathological examination using the light microscope.

#### 2.5.8. Statistical Analysis

In a behavior stress test, a percent (%) of change of movement count was calculated; it was considered 100% for all rats in all groups before doing the surgical intervention for all groups (base-line activity), then square root transformed % was calculated for % change from the normal base-line value, according to Jones et al. [46]. These calculations were done in order to avoid normal biological variations in the activity of normal rats in all groups (provided that each group contains rats with approximately similar activity). A comparison between more than two different groups was carried out using the non-parametric one-way analysis of variance ANOVA, followed by Dunn’s multiple comparisons test. Differences were considered significant at *p* < 0.05. Statistical analysis for hot plate and the biochemical parameters were done using ANOVA, followed by Tukey Kramer’s multiple comparisons test. Differences were considered significant at *p* < 0.05 using GraphPad Prism V.6.0.

## 3. Results and Discussion

### 3.1. Particle Size Analysis, Polydispersity Index (PDI) and Zeta-Potential

In order to obtain more precise information about the particle size, their distribution, and zeta potential, a Malvern zetasizer (ver. 6.20, Malvern, UK) was used. The mean particle size, PDI, and zeta potential of the different prepared mixed micelles of geraniol are cited in Table 1. Results reveal that all of the prepared geraniol mixed micelles formulae have a considerable small particle size with mean value ranged from 30.70 ± 1.42 to 102.36 ± 0.51 nm.

**Figure 1:**
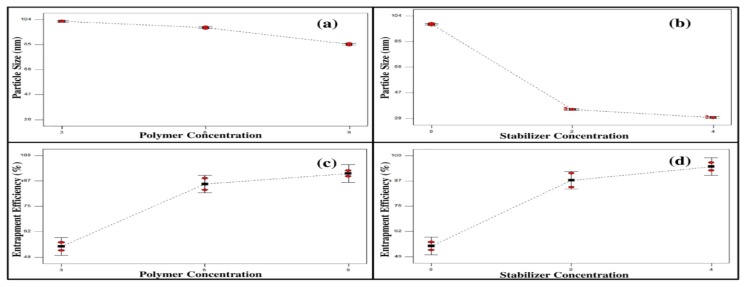
Line charts showing the effect of polymer and stabilizer concentration on the particle size (**a**,**b**) and the entrapment efficiency (**c**,**d**) of the prepared mixed micelle formulae.

Figure 1a shows the effect of polymer concentration on the particle size where a significant decrease (*p* < 0.0001) in the particle size was observed upon increasing polymer concentration. It seems that the mean size of the micelles was inversely related to the polymer content [47]. This might be attributed to the surfactants property of the polymer used, Pluronic^®^ F127, which allows the formation of smaller droplets by increasing the interfacial stability of polymeric mixed micelles [48].

Upon studying the effect of stabilizer (Cremophor EL) concentration (0%, 2%, and 4% *w/w*) on the particle size, it was observed that increasing the concentration of stabilizer resulted in decreasing the particle size significantly (*p* < 0.0001), as illustrated in Figure 1b. This might be reckoned to the presence of large number of surfactant molecules at the interfacial layer, which resulted in decreasing the surface tension and therefore promoting the formation of smaller droplets [49,50,51].

The PDI value was ranged from 0.198 ± 0.005 to 0.479 ± 0.061, indicating the homogeneity of the preparations. Zeta potential is a key factor to evaluate the stability of diluted micelles, whether its value is positive or negative, as it allows predicting good stability due to the high-energy barrier between particles and is influenced by its composition and the nature of medium [52]. The zeta potential of geraniol mixed micelles was found to range from −7.50 ± 3.62 to −20.8 ± 3.76 mV; this negative charge is attributed to the presence of geraniol in the mixed micelle systems [53].

### 3.2. Determination of Drug Loading (DL) and Encapsulation Efficiency (EE)

The drug loading of the different prepared mixed micelle formulae varied from 15.35 ± 0.99% to 32.85 ± 1.45% while the entrapment efficiency varied from 54.36 ± 2.85% to 97.85 ± 1.90%, as shown in Table 1. It is obvious that the %EE is directly proportional to the polymer and stabilizer concentrations, as illustrated in Figure 1c,d.

It is clear that increasing the polymer concentration and stabilizer concentration in the prepared mixed micelles resulted in increasing the %EE significantly, *p* < 0.0028 and *p* < 0.0002, respectively. This could be explained on the basis that the decrease in the polymeric size of nanoparticles upon increasing the polymer or stabilizer concentrations causes the surface area to increase, leading in turn to the increase in the drug entrapment efficiency [49,54].

### 3.3. In-Vitro Release Study

**Figure 2:**
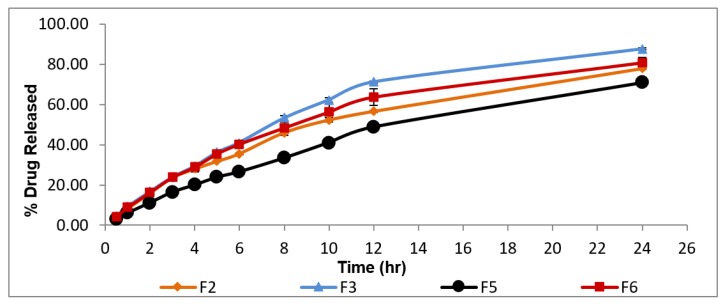
In-vitro release profile of geraniol from the selected mixed micelles F2, F3, F5, and F6 (±S.D, n = 3).

Design Expert^®^ software was used to investigate the desirability values of the different prepared formulae. Two sets of factors contributed in the choice of the desirable formulae, namely, maximum values of %EE and minimum values of particle size. Therefore, formulae F3, F6, F5, and F2 were selected for in-vitro release study where they showed the highest desirability values (0.96, 0.94, 0.88, and 0.80, respectively). The in-vitro release profile of geraniol from selected formulae is illustrated in Figure 2.

Upon increasing Cremophor El concentration (Table 1), the percentage release efficiency (%RE) of geraniol increased significantly, *p* < 0.05. This might be attributed to the decrease in the diffusion path lengths and the increase in the surface area as a result of particle size decreasing [55]. In addition, the penetration enhancing effect of Cremophor El increases the fluidity of the lipid bilayer [28,56].

On the other hand, increasing the concentration of Pluronic^®^ F127 resulted in decreasing the percentage RE of geraniol. This could be explained on the basis that it has a high molecular weight which means more abundance of O and OH points that enhance attachment to the drug molecule via hydrogen bonds leading to slower release rate [57]. Based on the previously discussed results, F3 which is composed of high concentration of Cremophor El and low concentration of Pluronic^®^ F-127 possessing the highest desirability value (0.96) was selected as the most desirable mixed micelles for further investigation in the current research.

### 3.4. Fourier Transform Infrared Spectroscopy (FTIR)

**Figure 3:**
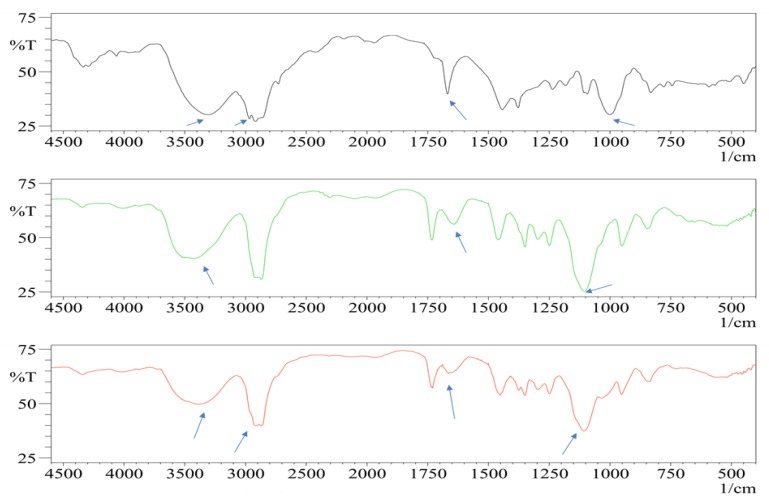
FTIR spectrum of free form geraniol oil, plain Formula F3, and polymeric mixed micelles Formula F3 (from top to bottom). The arrows show beak intensities changes and shifts.

Geraniol oil, 2,6-Dimethyl-2,6-octadien-8-ol, shows its characteristic peaks at 3306 and 1669 cm^−1^ [58]. These peaks correspond to O–H and C=C, respectively. In the plain formula, several peaks can be distinguished at 3500, 1750, and 1107 cm^−1^ owing to O–H, C=O, and C–H groups, respectively, which are corresponding to the formula components main active groups of Cremophor EL and Pluronic F127 [59,60]. In the case of geraniol polymeric mixed micelles formula (F3), changes of absorption bands intensities and peak shift at 3372, 2973, 2669, and 980 cm^−1^ can be easily distinguished. Such changes suggested that the functional groups of geraniol oil were included and interacted with the formulation functional groups. Thus, geraniol oil was successfully encapsulated inside the prepared mixed micelles [31,58], as shown in Figure 3.

### 3.5. Transmission Electron Microscope

**Figure 4:**
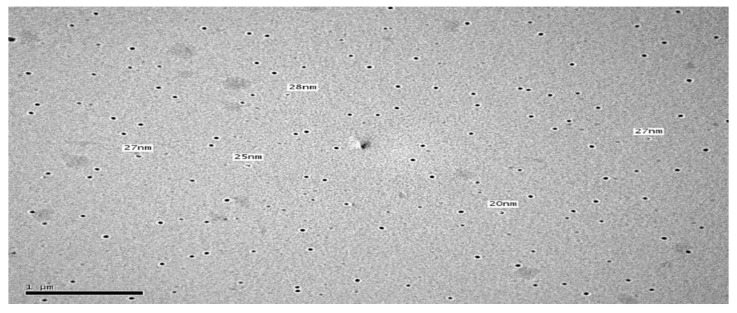
TEM photomicrograph of geraniol mixed micelles (F3).

To study the morphology of the optimal formulation (F3) and confirm the results obtained from Malvern particle size analyzer, TEM examination was done. The TEM image of F3 is illustrated in Figure 4 and revealed that all micelles were spherical in shape with no aggregation with good dispersibility. In addition, the mean size of the micelles was in good agreement with the size obtained from the Malvern particle size analyzer.

### 3.6. Pharmacological Study

The doses of micelles and oil that were used in the present prophylactic and therapeutic studies of the effect of geraniol on brain injury were selected based on the acute toxicity study. Brain injury caused by ischaemia/reperfusion (I/R) results in permanent handicapping, as observed in the behavior stress tests done in the present study (Table 2, Table 3 and Table 4). I/R exerts its deleterious effect via oxidative damage of proteins, membrane lipids, and nucleic acids. Low levels of tissue antioxidants associated with an increase in lipid peroxidation that have been demonstrated in animal models of brain ischaemia. I/R injury also promotes local inflammatory response involving microglial activation [61].

**Figure 5:**
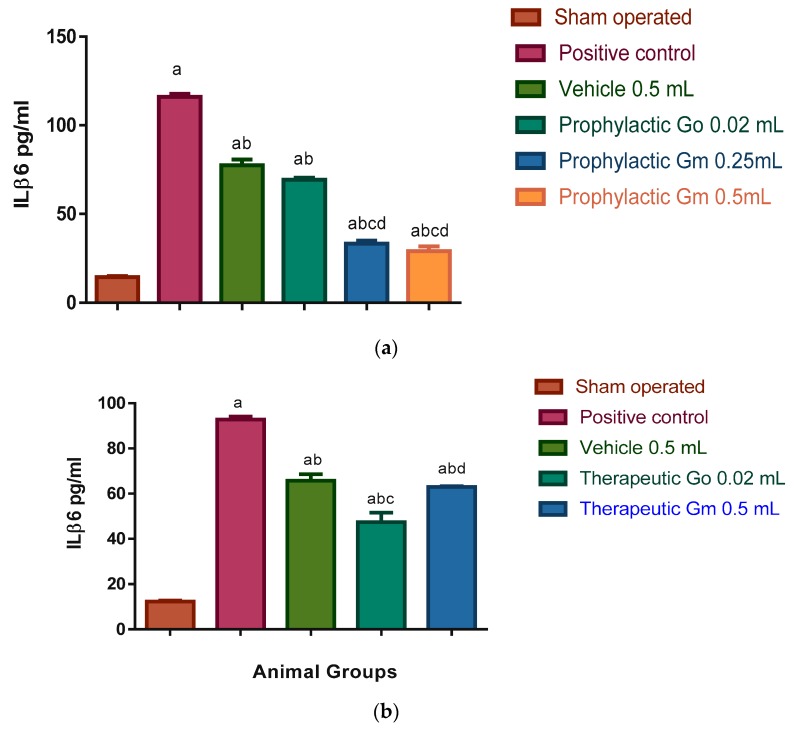
(**a**) Prophylactic effect of geraniol micelles vs. oil on ILb6 level in sera of I/R rat models (N = 8). Results were expressed as means of ILb6 level in sera ± S.E. Go: geraniol oil; Gm, geraniol micelles; (a) significantly different from sham-operated group; (b) significantly different from positive control group; (c) significantly different from vehicle group (0.5 mL); (d) significantly different from prophylactic geraniol oil (0.02 mL) group. (**b**) Therapeutic effect of geraniol micelles vs. oil on ILb6 level in sera of I/R rat models (N = 8). Results were expressed as means of ILb6 level in sera ± S.E. Go: geraniol oil; Gm, geraniol micelles; (a) significantly different from sham-operated group; (b) significantly different from positive control group; (c) significantly different from vehicle group (0.5 mL); (d) significantly different from group treated with geraniol micelles (0.5 mL).

**Figure 6:**
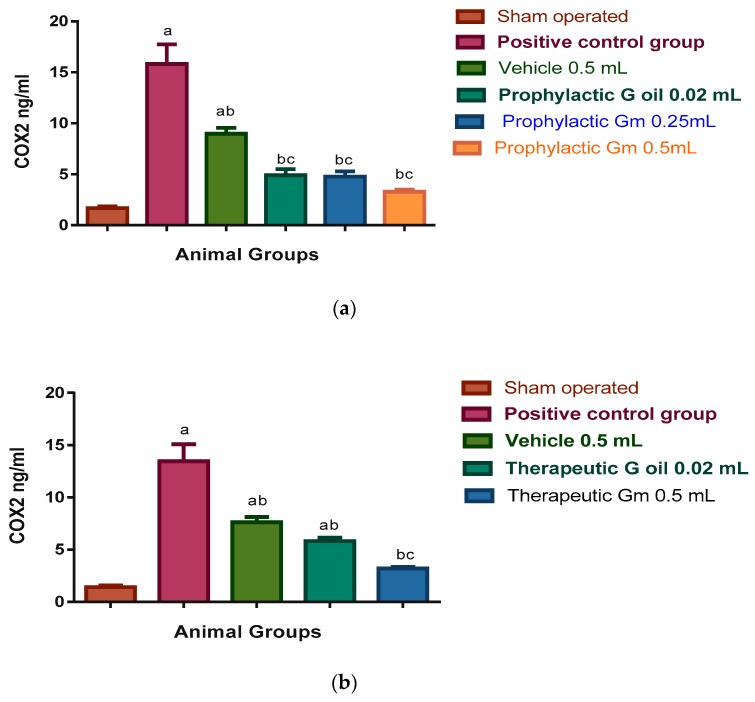
(**a**) Prophylactic effect of geraniol micelles vs. oil on COX-2 level in sera of I/R rat models (N = 8). Results were expressed as means of COX-2 level in sera ± S.E. Go: geraniol oil; Gm: geraniol micelles; (a) significantly different from sham-operated group; (b) significantly different from positive control group; (c) significantly different from vehicle group. (**b**) Therapeutic effect of geraniol micelles vs. oil on COX-2 level in sera of I/R rat models (N = 8). Results were expressed as means of COX-2 level in sera ± S.E. Go: geraniol oil; Gm: geraniol micelles; (a) significantly different from sham-operated group; (b) significantly different from positive control group; (c) significantly different from vehicle group.

**Figure 7:**
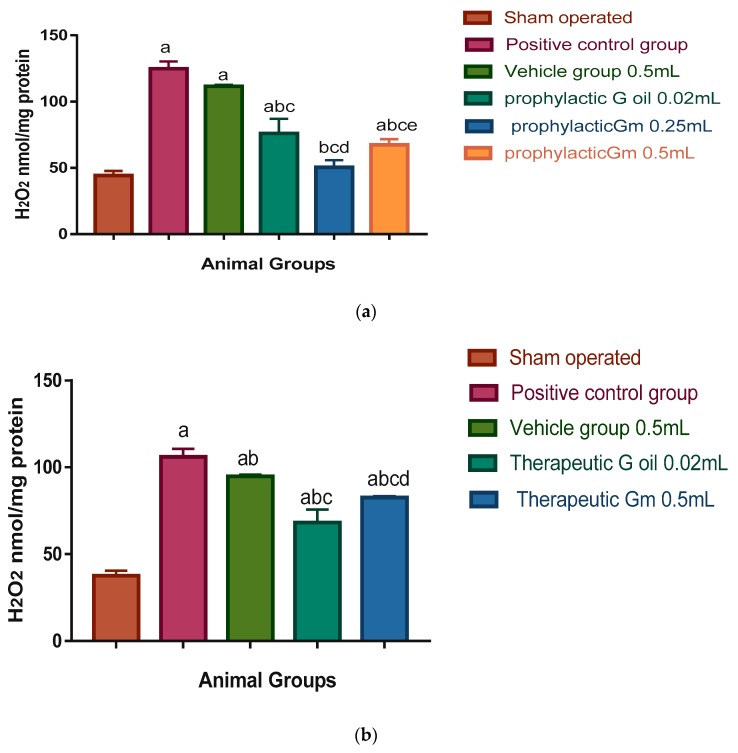
(**a**) Prophylactic effect of geraniol micelles vs. oil on H_2_O_2_ level in brain homogenate of I/R rat models (N = 8). Results were expressed as means of H_2_O_2_ level in brain homogenate ± S.E. Go: geraniol oil; Gm: geraniol micelles; (a) significantly different from sham-operated group; (b) significantly different from positive control group; (c) significantly different from vehicle group; and (d) significantly different from group treated with geraniol oil (0.02 mL); (e) significantly different from group treated with geraniol micelles (0.25 mL). (**b**) Therapeutic effect of geraniol micelles vs. oil on H_2_O_2_ level in brain homogenate of I/R rat models (N = 8). Results were expressed as means of H_2_O_2_ level in brain homogenate ± S.E. Go: geraniol oil; Gm: geraniol micelles; (a) significantly different from sham-operated group; (b) significantly different from positive control group; (c) significantly different from vehicle group; and (d) significantly different from group treated with geraniol oil (0.02 mL).

**Figure 8:**
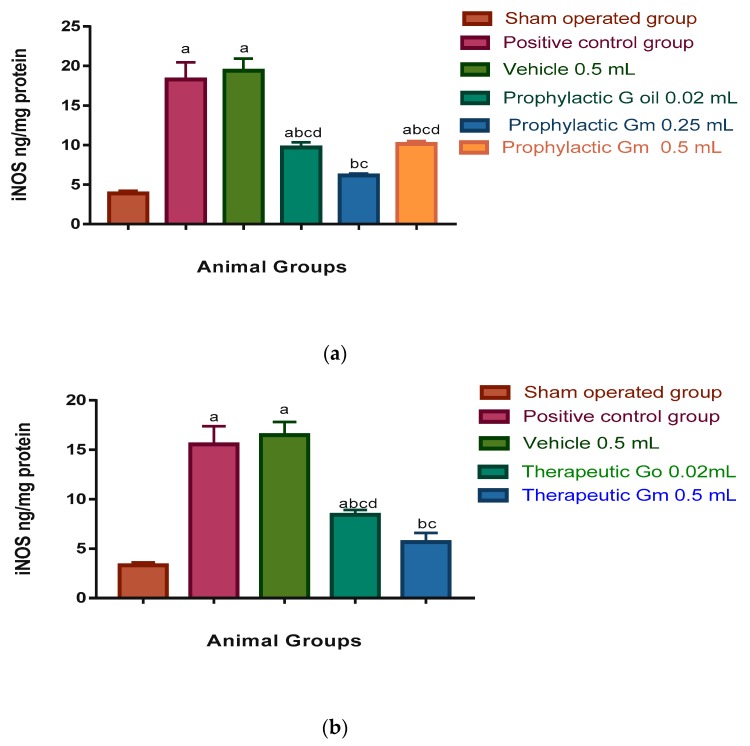
(**a**) Prophylactic effect of geraniol mesticles vs. oil on NOS level in brain homogenates of I/R rat models (N = 8). Results were expressed as means of NOS level in brain homogenates ± S.E. Go: geraniol oil; Gm: geraniol micelles; (a) significantly different from sham-operated group; (b) significantly different from positive control group; (c) significantly different from vehicle (0.5 mL) group; (d) significantly different from group prophylactic low dose of micelles (0.25 mL). (**b**) Therapeutic effect of geraniol mesticles vs. oil on NOS level in brain homogenates of I/R rat models (N = 8). Results were expressed as means of NOS level in brain homogenates ± S.E. Go: geraniol oil; Gm: geraniol micelles; (a) significantly different from sham operated group; (b) significantly different from positive control group; (c) significantly different from vehicle (0.5 mL) group; (d) significantly different from group therapeutic dose of micelles (0.5 mL).

Microglial cell activation leads to the expression of cyclooxygenase-2 (COX-2) and inducible nitric oxide synthase (iNOS), which are the proinflammatory enzymes. Also, increased permeability of cerebral capillaries due to ischaemia and post-ischaemic reperfusion results in oedema and haemorrhage which are deleterious for brain tissue [62]. These findings are consistent with significant elevation of inflammatory markers ILβ6 (116.03 and 92.83 pg/mL in prophylactic and therapeutic groups respectively); COX-2 (15.82 and 13.45 ng/mL in prophylactic and therapeutic groups, respectively); H_2_O_2_ (124.7 and 106.06 nmol/mg protein in prophylactic and therapeutic groups, respectively); and iNOS (18.3 and 15.55 ng/mg protein in prophylactic and therapeutic groups, respectively) of the positive control group, when compared to the sham-operated group where the levels of inflammatory markers were as follows: ILβ6 (14.43 and 12.25 pg/mL in prophylactic and therapeutic groups, respectively); COX-2 (1.68 and 1.42 ng/mL in prophylactic and therapeutic groups, respectively); H_2_O_2_ (44.32 and 37.67 nmol/mg protein in prophylactic and therapeutic groups, respectively); and iNOS 3.9 and 3.31 ng/mg protein in prophylactic and therapeutic groups, respectively; see Figure 5, Figure 6, Figure 7 and Figure 8).

These also explain the deleterious effect on behavior observed in the positive control group when examined by using a grid floor activity cage to test the psychological state; the movements of rats of both positive control and vehicle (plain micelles) group were zero when assessed at 24 and 72 h after I/R (Table 2). Also, locomotor coordination tested by rotarod (Table 3) and sensation tested by hot plate (Table 4) were zero at 72 h after I/R due to hind limb paralysis and forelimb paresis of rats in both groups in the prophylactic study, which led to loss of sensation and inability of animals to maintain balance on the accelerating rotarod, while in therapeutic study, behaviour and sensation could not be assessed for both groups after induction of ischaemia due to the same reasons, in addition to animal deaths.

That is why the present study was conducted to evaluate the prophylactic and therapeutic effects of the novel drug delivery system (DDS) in the form of geraniol mixed micelles that targeted the central nervous system (CNS), as was proven by behavioral stress tests.

Testing the psychological state using the grid floor activity cage at zero time before starting the experiment for all animals in all groups, then treatment vehicle (plain micelles 0.5 mL), geraniol oil (0.02 mL), and geraniol micelles (0.25 and 0.5 mL) was given intra-nasally for 72 h before I/R induction by left CCA ligation in order to achieve an adequate level of tested substances. Results expressed in Table 2 show that almost all animals at zero time “base-line record” exhibited more or less the same number of movements/5 min as recorded by number of oscillations of the grid floor of the activity cage.

Seventy-two hours after the instillation of vehicle (plain micelles) and geraniol, either oil or micelles, in both doses, there was a significant decrease in the activity of treated groups only compared to the sham-operated group and positive control groups prior to induction of I/R. This is most probably due to the sedating effect of geraniol which was reported by Medeiros et al. [63], who demonstrated that geraniol had a depressant effect on the CNS when given in doses of 50 and 100 mg/kg. That is why also during the examination of the analgesic effect of the micelles and oil by using the hot plate, the time elapsed before licking the hind limb increased when rats were intranasally administered geraniol prior to I/R, demonstrating the sedating and analgesic effects of geraniol.

On the other hand, the activity of group receiving the high dose increased after 144 h (72 h after I/R) of geraniol administration and the time elapsed before licking the hind paw in the hot plate test decreased most probably due to tolerance to geraniol, as most cases with agents that have depressant effects on the CNS.

Yet the movements of rats treated with either geraniol oil or low dose micelle were significantly less than the sham-operated group and the group treated with micelles in high doses. It is noteworthy that the movements of rats of the sham-operated group in the grid floor activity cage, as well as their duration of balance on the rotarod, were not significantly changed throughout the experiment period. Also, their sensation and response to pain were nearly the same throughout the experiment.

A locomotor co-ordination test was done by using an accelerating rotarod; results expressed in Table 3 show that all rats in all groups could maintain balance on the accelerating speed rotarod for 180 s at zero time “base-line record”. The balance of positive control vehicle group and micelle high dose groups was maintained at 180 s after 72 h of treatment prior to I/R, while that of the groups that received the oil or micelle in low dose IN decreased significantly in comparison to the other four groups, due to the previously mentioned CNS depressing effect of geraniol.

Twenty-four hours after I/R associated with IN treatment, the balance duration of the oil group was significantly less than that of the sham-operated and high dose micelle groups and significantly more than that of the low dose micelle group. While after 72 h of I/R associated with IN treatment the duration of balance of both oil and high dose micelle groups were the same, but it was significantly less than the sham-operated group and more than the low dose micelle group, which showed the least duration of balance during the experiment.

Deng et al.’s results confirmed our findings as they reported that geraniol given to mice for three weeks ameliorated the depression-like symptoms which rose as a result of mild stress. The results of their study showed that geraniol could improve the psychological and locomotor activities of rodents, which are the same as results of the present study; however, our study utilized intranasal route as a new DDS which involves shorter duration of administration to avoid long term side effects such as sedation [64].

The analgesic effect of the micelles and oil was tested by “Thermal test” using an electronic “Hot plate”. Results expressed in Table 4 show that after 24 h of I/R associated with IN treatment, the elapsed time before licking the hind paws of the positive control group increased significantly in comparison to the sham-operated, vehicle (plain micelles), and geraniol groups. The time of geraniol treated groups (oil and micelles) was significantly higher than the vehicle group. The micelles groups (high and low dose) were significantly higher than the oil group. At the same time, the high dose micelle analgesic effect was significantly higher than the sham-operated and low dose groups.

Seventy-two hours after I/R, the elapsed time before licking the hind paws of the positive control and vehicle groups dropped to zero due to inability to elevate the hind limbs due to paralysis and accompanying paraesthesia, while the elapsed time of oil and low dose micelle groups increased significantly in comparison to the sham-operated, positive control, vehicle, and high dose micelle groups. The low dose micelle group onset of pain sensation increased significantly in comparison to the sham-operated, oil and high dose micelle groups. The high dose micelle group onset of pain sensation decreased significantly in comparison to the sham-operated and oil groups.

Results of ILβ6 expressed in Figure 5a show significant elevation of its levels in vehicle (plain micelles), prophylactic geraniol oil (0.02 mL), prophylactic geraniol micelles (0.25 mL) as it was 77.4, 69.36, and 33.26 pg/mL, respectively, in comparison to the sham-operated group (14.43 pg/mL), while the level of ILβ6 in prophylactic geraniol micelle (0.5 mL) group was 29 pg/mL which was not significantly different from the sham-operated group. All groups showed a significant reduction in comparison to positive control group (116.03 pg/mL).

Also, results expressed in Figure 5b show its elevation in vehicle, therapeutic geraniol oil (0.02 mL), and therapeutic geraniol micelle (0.5 mL): 65.78, 47.4, and 63.03 pg/mL, respectively, in comparison to the sham-operated group (12.25 pg/mL) and significant reduction in comparison to positive control group (92.83 pg/mL).

As for results of COX-2 of the prophylactic groups expressed in Figure 6a, the levels of COX-2 in sera of vehicle group, geraniol oil (0.02 mL), geraniol micelle (0.25 mL), geraniol micelle (0.5 mL), namely, 8.97, 4.9, 4.77, and 3.29 ng/mL, respectively, were significantly less than the positive control group (15.82 ng/mL), while only the vehicle group was significantly more than the sham-operated group (1.68 ng/mL). All geraniol treated groups showed significant reduction in comparison to the vehicle group.

Regarding the results of the therapeutic groups expressed in Figure 6b, the levels of COX-2 in sera of the vehicle group, geraniol oil (0.02 mL), geraniol micelle (0.5 mL), namely, 7.62, 5.82, and 3.2 ng/mL, respectively, were significantly less than the positive control group (13.45 ng/mL), while the vehicle and oil groups were significantly more than the sham-operated group (1.42 ng/mL). The geraniol micelles treated group showed a significant reduction in comparison to the vehicle group.

Figure 7a shows that H_2_O_2_ levels were significantly elevated in brain homogenates of the vehicle and prophylactic geraniol oil groups and prophylactic geraniol micelles group (0.5 mL) (111.6, 76.05, and 67.46 nmol/mg protein, respectively), in comparison to the sham-operated group (44.32 nmol/mg protein). On the other hand, the levels of H_2_O_2_ in homogenates of prophylactic geraniol oil (0.02 mL), prophylactic geraniol micelle (0.25 and 0.5 mL), were 76.05, 50.46, and 67.46 nmol/mg protein, respectively, which were significantly less than the positive control and vehicle groups (124.78 and 111.6 nmol/mg protein, respectively). The level of H_2_O_2_ in homogenates of prophylactic low geraniol micelle dose (0.25 mL) was significantly less than the prophylactic geraniol oil and high geraniol micelle dose (0.5 mL).

Moreover, Figure 7b shows that the levels of H_2_O_2_ in brain homogenates of the therapeutic vehicle, therapeutic geraniol oil (0.02 mL), and therapeutic geraniol micelle (0.5 mL) groups were significantly more than the sham operated group (94.86, 68.13, 82.6, and 37.67 nmol/mg protein, respectively). However, all groups were significantly more than the positive control group (106.06 nmol/mg protein).

When iNOS was measured in brain homogenates of rats of the prophylactic groups, the results expressed in Figure 8a showed a significant elevation of its level in the vehicle, prophylactic geraniol oil (0.02 mL), and prophylactic geraniol micelle (0.5 mL) groups (19.4, 9.72, and 10.15 ng/mg protein, respectively) in comparison to the sham-operated group whose level was 3.9 ng/mg protein. The levels of iNOS in brain homogenates of prophylactic geraniol oil (0.02 mL), prophylactic geraniol micelles (0.25 and 0.5 mL) were significantly less than the positive control group (18.3 ng/mg protein) and the vehicle group. The level of iNOS in the brains of the prophylactic low dose micelles group is significantly less than the levels in brains of the prophylactic oil and prophylactic high dose micelle groups.

As for the therapeutic study (Figure 8b), the levels of iNOS in brain homogenates of the vehicle (0.5 mL) and geraniol oil groups (0.02 mL), 16.49 and 8.4 ng/mg protein, respectively, were significantly more than the sham-operated group (3.31 ng/mg protein). Both the therapeutic geraniol oil group and the micelle (0.5 mL) group whose level was 5.65 ng/mg protein showed significantly less levels than the positive control group whose level was 15.55 ng/mg protein. Moreover, the level of iNOS in brain homogenates of the micelle group was significantly less than the oil group.

Rekha et al. have illustrated that geraniol exerted a neuro-protective in a rodent model of Parkinsonism due to its ability to cross the cell membrane and suppress lipid peroxidation and mRNA expressions [65]. Also, Rekha et al. in another study stated that administration of geraniol prior to the induction of Parkinsonism had improved neuromuscular malfunction and tyrosine hydroxylase expression in a dose dependant manner. In addition, it decreased α-synuclein expression [66].

In the study of Elmann et al., the major constituents of geraniol oil could not inhibit nitric oxide (NO), which is produced as a result of the activation of microglial cells, but they suggested that at higher concentrations, citronellol (which can be prepared by hydrogenation of geraniol) could inhibit NO activity, which represents an important neuro-inflammatory mediator involved in nerve cell apoptosis [67]. They concluded that geraniol oil might be effective in the prevention or even treatment of neurodegenerative diseases. The results of Elmann et al. explain the lowering effect of geraniol micelles of inflammatory markers in our study.

However, Su et al.’s results were in agreement with our result as they found that geraniol could inhibit NO production, together with a reduction in protein and mRNA expression levels of iNOS. Geraniol and citronellol additively reduced the LPS-induced COX-2 protein and mRNA expression [68].

### 3.7. Histopathologic Examination Results

The post-mortem histopathologic examination in the present study confirms the presence of inflammation in the form of thickened vascular pia mater, outer distorted grey matter, white matter, red neuron, oedema, and axonal spheroids in the cerebral cortex of the positive control group as illustrated in Figure S1. Besides, histopathologic examination showed that the frontal cortex of the sham group was covered by pia mater containing blood vessels. Six layers were identified in the cerebral cortex—outer molecular layer, external granular layer, external pyramidal layer, inner granular layer, inner pyramidal, and the polymorphic layer (Supplementary Figure S2).

Section of the cerebral cortices of the prophylactic group (vehicle group) showed thickened and vascular pia mater, oedma under pia, red neuron infarction, and many apoptotic cells (Supplementary Figure S3). Also, the photomicrograph of prophylactic geraniol oil group showed infarction in grey matter and white matter, many apoptotic and cellular infiltrations, and oedema (Supplementary Figure S4). While a section of the prophylactic geraniol micelle (0.25 mL) group showed infarction in small areas, mild oedema mainly in white matter and increased cellular infiltration (Supplementary Figure S5) and photomicrograph of the prophylactic geraniol micelle (0.5 mL) group showed pia mater with cellular infiltration, oedema, red neuronal infarction, cellular infiltration, apoptotic and mitotic cells (Supplementary Figure S6).

On the other hand sections of the therapeutic geraniol oil (0.02 mL) group showed thin pia, many infarct areas, high vascularity with congested blood vessels and haemorrhage, oedema and apoptotic cells (Supplementary Figure S7), while photomicrograph of therapeutic geraniol micelle (0.5 mL) showed areas of infarction, mild oedema in both grey matter and white matter and cellular infiltration (Supplementary Figure S8).

These results emphasize the neuro-protective effect of micelles due to the anti-inflammatory effect of micelles as they are capable of crossing the blood–brain barrier; they also show that the effect of micelles is dose dependant and that the prophylactic effect is much better than the therapeutic effect. This explains the prophylactic effect of high dose of polymeric mixed micelle in our study observed by improvement of signs and symptoms of I/R injury in the treated group compared to untreated positive control and oil groups as well as the effect of high dose of micelle on biochemical parameters when given post-I/R in the therapeutic study.

## 4. Conclusions

The results of studying the behaviour of animals by stress tests, examining the sensation by thermal hot plate test, assessing the anti-inflammatory effect of the novel geraniol polymeric mixed micelles by assay of Ilβ6, COX-2, H_2_O_2_, and iNOS in brain homogenates and finally post-mortem histopathologic examination of rat cortices, were all consistent with each other, and confirmed that geraniol in the form of nanostructured mixed micelles could be considered a promising long circulating intranasal nano-carrier prophylactic measure which could be used for prophylaxis against damage to brain cells induced by ischaemia and subsequent reperfusion.

Most evaluating tests performed in the present study suggest that the effect of the nanostructured mixed micelles is better than the oil effect and is dose dependant, which makes the optimized micelle formula a novel easy DDS. Moreover, it has predicted better bioavailability as intranasal route had rapid brain access and better effect due to its ability of bypassing the geraniol breakdown by liver that happens orally.

However, its therapeutic effect could not be confirmed by this study due to the short duration which the study regimen was directed as a result of most animal paralysis or death after the induction of I/R, which hindered the ability to perform behaviour stress tests or sensation evaluation.

## Figures and Tables

**Table 1:** The composition of the differently prepared geraniol mixed micelles formulae and their characterization tests.

Code	Composition (% *w/v*)		Zeta Potential (mV) (±S.D)	PDI (±S.D)	Mean Particle Size (nm) (± S.D)	Entrapment Efficiency (%) (±S.D)	Drug Loading (DL) (%) (±S.D)
	Stabilizer Concentration (Cremophor EL)	Polymer Concentration (Pluronic F127)					
F1	0	3	−20.00 ± 3.25	0.198 ± 0.005	102.36 ± 0.51	54.36 ± 2.85	27.18 ± 0.45
F2	2	3	−11.60 ± 1.17	0.381 ± 0.009	40.44 ± 1.37	87.6 ± 4.97	32.85 ± 1.45
F3	4	3	−20.80 ± 3.76	0.352 ± 0.013	32.46 ± 0.64	97.85 ± 1.90	29.35 ± 0.24
F4	0	6	−17.80 ± 0.81	0.238 ± 0.011	99.15 ± 2.45	85.68 ± 4.17	28.56 ± 0.50
F5	2	6	−19.50 ± 3.10	0.304 ± 0.006	34.72 ± 0.52	91.27 ± 0.79	24.89 ± 0.12
F6	4	6	−14.60 ± 2.17	0.235 ± 0.007	28.63 ± 0.40	93.58 ± 1.45	21.60 ± 0.32
F7	0	9	−7.50 ± 3.62	0.256 ± 0.004	86.13 ± 1.76	90.98 ± 1.98	22.75 ± 0.56
F8	2	9	−9.71 ± 3.26	0.479 ± 0.061	40.68 ± 0.71	87.46 ± 4.41	21.86 ± 1.09
F9	4	9	−9.19 ± 4.69	0.302 ± 0.039	30.70 ± 1.42	81.87 ± 6.41	15.35 ± 0.99

All of the prepared mixed micelles contain 3% geraniol. Data are presented as mean average value (±S.D, n = 3).

**Table 2:** Behavioral stress test done by using a grid floor activity cage to evaluate the prophylactic effect of geraniol micelles on psycho-motor state insult induced by ischaemia/reperfusion injury in rats.

	Onset	Groups					
		Sham Operated	Positive Control	Vehicle (0.5 mL)	Geraniol Oil (0.02 mL)	Geraniol Micelles (0.25 mL)	Geraniol Micelles (0.5 mL)
**Pre-Ischaemic/Reperfusion geraniol ttt**	Base-line (Normal rats)	1	1	1	1	1	1
	72 h (once/24 h × 3 days)	0.99 ± 0.02	0.99 ± 0.02	0.63 ^ab^ ± 0.01	0.62 ^ab^ ± 0.01	0.61 ^ab^ ± 0.04	0.7 ^abcde^ ± 0.03
**Post-Ischaemic/Reperfusion geraniol ttt**	24 h	0.98 ± 0.01	0 ^a^	0.13 ^abd^ ± 0.02	0.39 ^abc^ ± 0.01	0.23 ^abcd^ ± 0.02	0.28 ^abcd^ ± 0.01
	72 h (once/24 h × 3 days)	0.99 ± 0.01	0 ^a^	0^a^	0.11 ^abc^ ± 0.006	0.16 ^abc^ ± 0.02	0.5 ^abcde^ ± 0.02

N = 8, results were expressed as means of square root transformed % of number of movements/5 min ± S.E. (a) means significantly different from the sham-operated group; (b) significantly different from positive control (I/R) group; (c) significantly different from vehicle (0.5 mL) group; (d) significantly different from prophylactic geraniol oil (0.02 mL) group, and (e) significantly different from prophylactic geraniol micelles (0.25 mL) group.

**Table 3:** Behavioral stress test done by using a rotarod to evaluate the prophylactic effect of geraniol micelles on locomotor deficit that is induced by ischaemia/reperfusion injury in rats.

	Onset	Groups					
		Sham Operated	Positive Control	Vehicle (0.5 mL)	Geraniol Oil (0.02 mL)	Geraniol Micelles (0.25 mL)	Geraniol Micelles (0.5 mL)
**Pre-Ischaemic/** **Reperfusion** **Geraniol ttt**	Base-line (Normal rats)	180	180	180	180	180	180
	72 h (once/24 h × 3 days)	180	180	180	120 ^abc^ ± 4.08	113.7 ^abc^ ± 4.2	180 ^de^
**Post-Ischaemic/** **Reperfusion** **Geraniol ttt**	24 h	180	0^a^	21.25 ^ab^ ± 1.49	120 ^abc^ ± 3.5	45 ^abcd^ ± 2.04	180 ^bcde^
	72 h (once/24 h × 3 days)	180	0^a^	0 ^ade^	90 ^abc^ ± 4	18 ^abcd^ ± 0.81	90 ^abce^ ± 3.53

N = 8, results were expressed as means of seconds maintained by rat on rotarod ± S.E. (a) means significantly different from sham operated group; (b) significantly different from positive control (I/R)group; (c) significantly different from vehicle (0.5 mL) group; (d) significantly different from prophylactic geraniol oil (0.02 mL) group; and (e) significantly different from prophylactic geraniol micelles (0.25 mL) group.

**Table 4:** Thermal test done by using a hot plate for evaluation of the analgesic effect of geraniol micelles when it is used for prophylaxis from ischaemia/reperfusion injury in rat models.

		Groups					
	Onset	Sham Operated	Positive Control	Vehicle (0.5 mL)	Geraniol Oil (0.02 mL)	Geraniol Micelles (0.25 mL)	Geraniol Micelles (0.5 mL)
**Pre-Ischaemic/Reperfusion** **geraniol ttt**	Base-line (Normal rats)	26.4 ± 1.29	26.4 ± 1.29	26.4 ± 1.29	26.4 ± 1.29	26.4 ± 1.29	26.4 ± 1.29
	72 h (once/24 h × 3 days)	28.4 ± 0.49	28.4 ± 0.49	21.8 ^ab^ ± 0.92	59.2 ^abc^ ± 0.72	65.8 ^abcd^ ± 1.19	58.3 ^abce^ ± 2.75
**Post-Ischaemic/Reperfusion** **Geraniol ttt**	24 h	31.4 ± 0.66	75 ^a^ ± 0	23.2 ^ab^ ± 1.68	33.4 ^bc^ ± 0.34	29.2 ^bcd^ ± 1.9	35.8 ^abce^ ± 0.59
	72 h (once/24 h × 3 days)	30.5 ± 0.64	0 ^a^	0^a^	39.4 ^abc^ ± 0.62	56.2 ^abcd^ ± 0.49	15.4 ^abcde^ ± 0.25

N = 8, results were expressed as means of seconds elapsed before licking the paw ± S.E. (a) means significantly different from sham-operated group; (b) significantly different from positive control (I/R) group; (c) significantly different from vehicle (0.5 mL) group; (d) significantly different from prophylactic geraniol oil (0.02 mL) group and (e) significantly different from prophylactic geraniol micelles (0.25 mL) group.

## Supplementary Materials

The following are available online at https://www.mdpi.com/1999-4923/12/1/76/s1, Figure S1: A photomicrograph of a section of cerebral cortex of the positive control group showing thickened vascular pia mater (arrow), outer distorted grey matter (GM), white matter (WM), red neuron (red arrow), oedema (star) and axonal spheroids (shape). Figure S2: A photomicrograph of a section of cerebral cortex of the sham group showing delicate layer of pia mater (arrow), outer grey matter six layers (GM) of cerebral cortex. Figure S3: Photomicrograph of the prophylactic vehicle group showed thickened and vascular pia mater (arrow), oedma under pia (star), red neuron infarction (red arrow), and many apoptotic cells. Figure S4: Photomicrograph of the prophylactic geraniol oil group showed infarction (green arrow) in grey matter (GM) and white matter (WM), many apoptotic and cellular infiltration (red arrow), oedema (stars). Figure S5: Photomicrograph of the prophylactic geraniol micelle (0.25 mL) group shows infarction in small areas (red arrow), mild edema (star) mainly in white matter (WM) and increased cellular infiltration (green arrows). Figure S6: Photomicrograph of the prophylactic geraniol micelle (0.5 mL) group showing pia mater with cellular infiltration, oedema (stars) red neuronal infarction (red arrow), cellular infiltration (rectangle), apoptotic and mitotic cells (green arrow). Figure S7: Photomicrograph of therapeutic geraniol oil (0.02 mL) showing thin pia (black arrow), many infarct areas (red arrows), high vascularity with congested blood vessels (BV) and haemorrhage (green arrow), oedema (star) note apoptotic cells (shape). Figure S8: Photomicrograph of therapeutic geraniol micelle (0.5 mL) showing areas of infarction (red arrow), mild oedema (star) in both GM and WM and cellular infiltration (green arrows).
