# Supplementary material for: Novel Intranasal Drug Delivery: Geraniol Charged Polymeric Mixed Micelles for Targeting Cerebral Insult as a Result of Ischaemia/Reperfusion
Source: Pharmaceutics. 2020 Jan 17;12(1):76. doi: 10.3390/pharmaceutics12010076 (PMC7022886; doi:10.3390/pharmaceutics12010076)
Supplement: Supplementary file 1 [file pharmaceutics-12-00076-s001.zip › Figure S8.pdf]

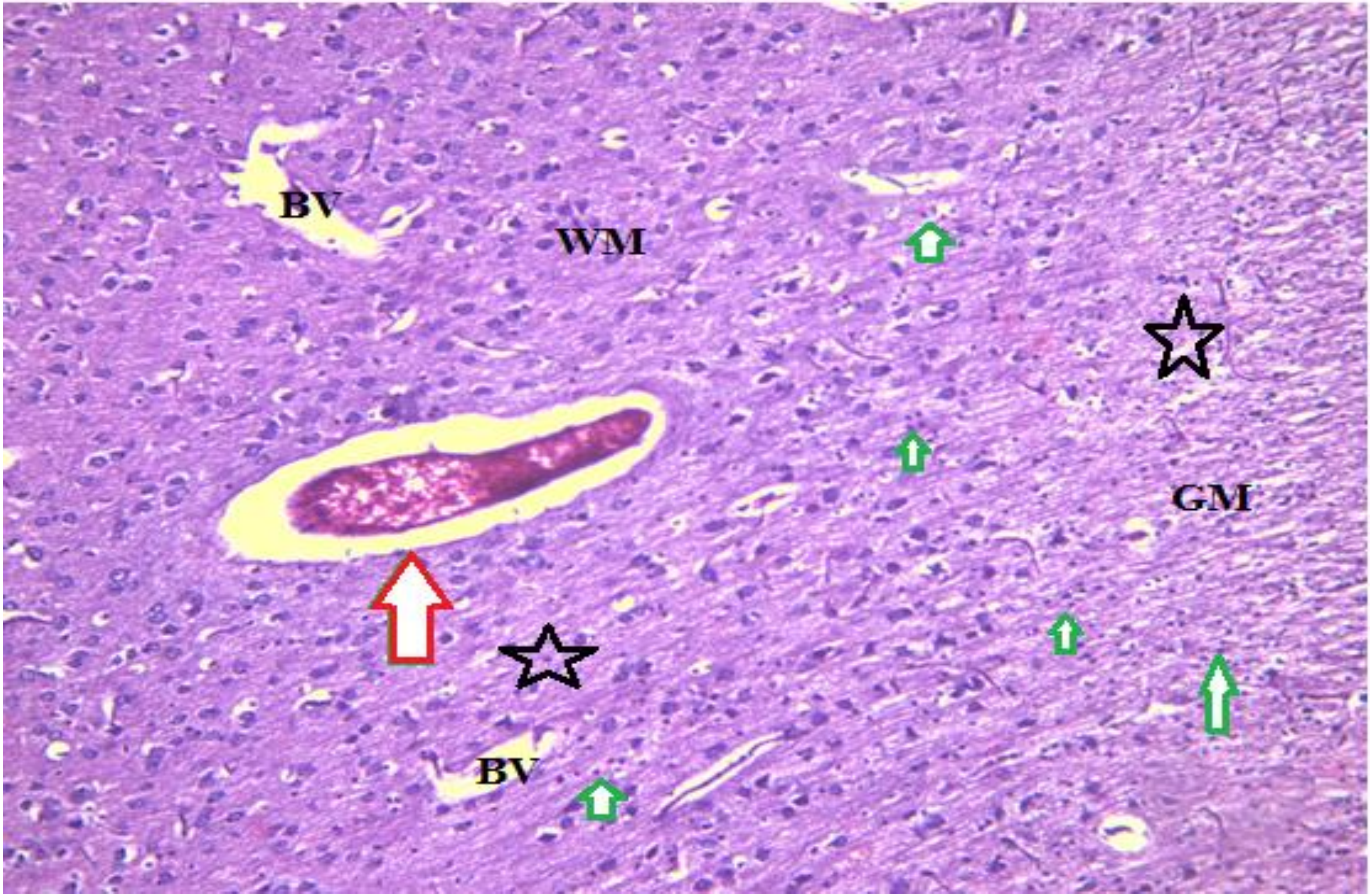

Figure S8. Photomicrograph of therapeutic Geraniol micelle (0.5ml) showing areas of infarction (red arrow), mild oedema (star) in both GM and WM and Cellular infiltration (green arrows)
