# Supplementary material for: Novel Intranasal Drug Delivery: Geraniol Charged Polymeric Mixed Micelles for Targeting Cerebral Insult as a Result of Ischaemia/Reperfusion
Source: Pharmaceutics. 2020 Jan 17;12(1):76. doi: 10.3390/pharmaceutics12010076 (PMC7022886; doi:10.3390/pharmaceutics12010076)
Supplement: Supplementary file 1 [file pharmaceutics-12-00076-s001.zip › Figure S1.pdf]

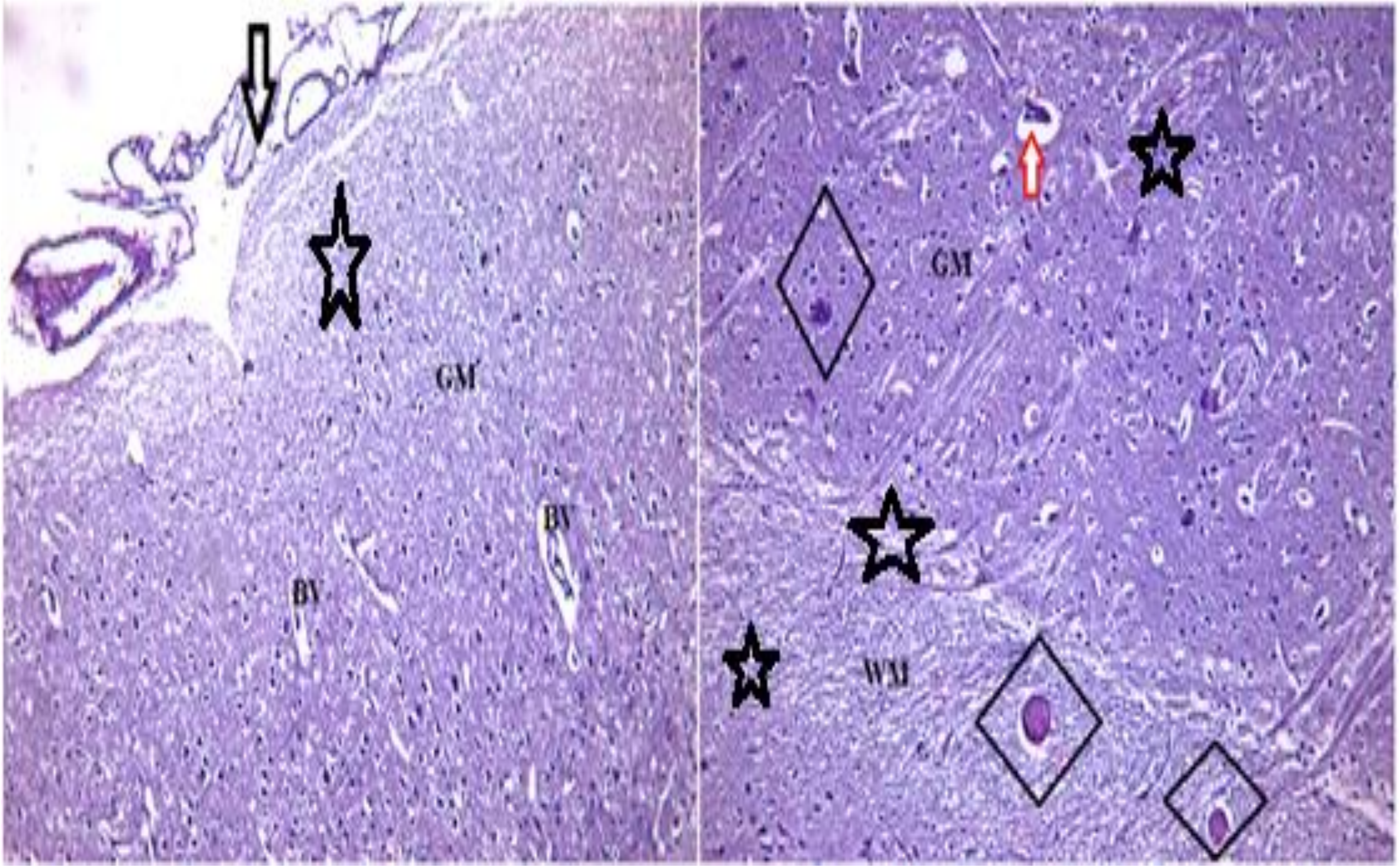

Figure S1. A photomicrograph of a section of cerebral cortex of positive control group showing thickened vascular pia matter (arrow), outer distorted grey matter (GM), white matter (WM), red neuron (red arrow), oedema (star) and axonal spheroids (shape)
