# Supplementary material for: Novel Intranasal Drug Delivery: Geraniol Charged Polymeric Mixed Micelles for Targeting Cerebral Insult as a Result of Ischaemia/Reperfusion
Source: Pharmaceutics. 2020 Jan 17;12(1):76. doi: 10.3390/pharmaceutics12010076 (PMC7022886; doi:10.3390/pharmaceutics12010076)
Supplement: Supplementary file 1 [file pharmaceutics-12-00076-s001.zip › Figure S2.pdf]

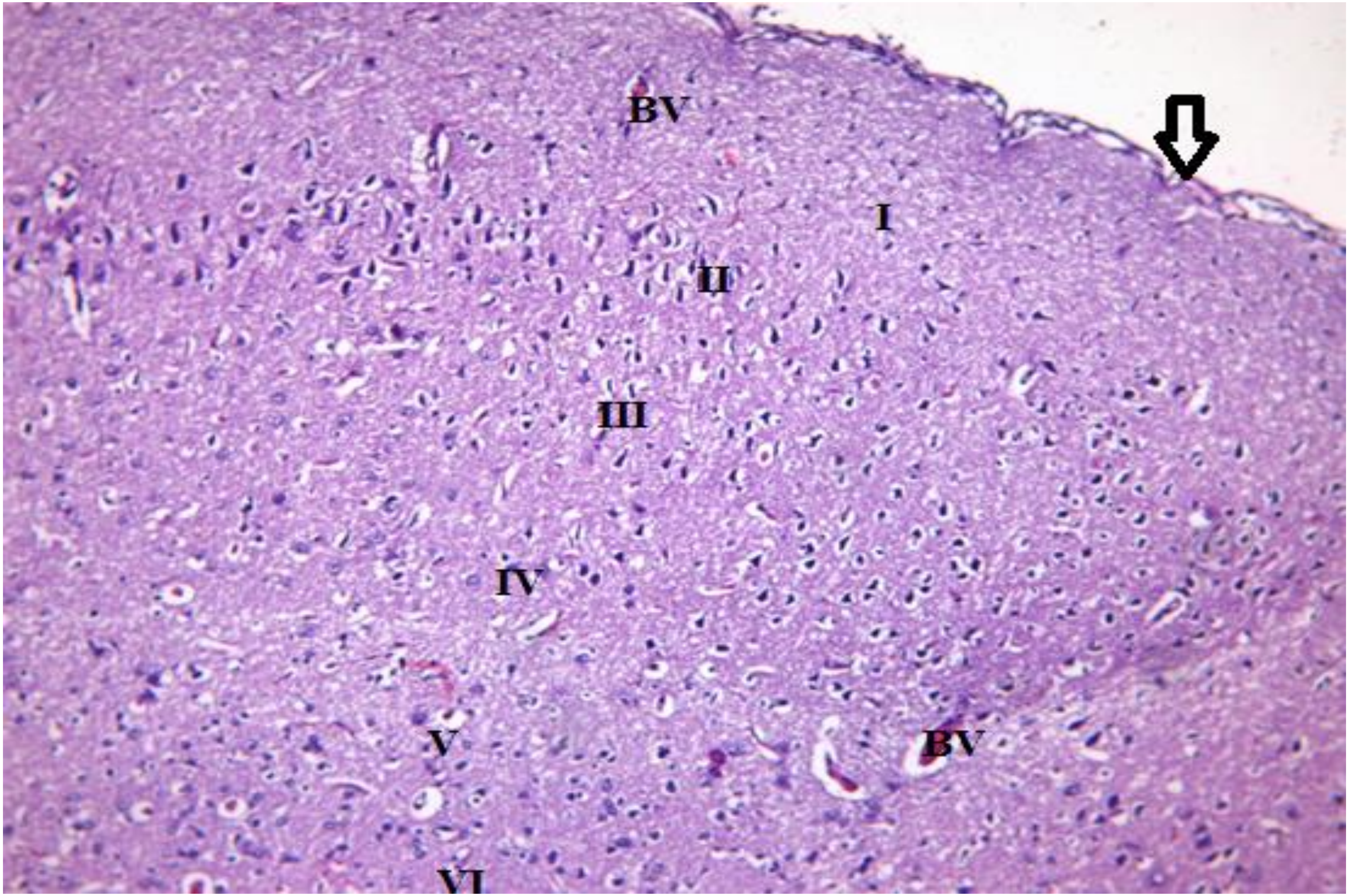

Figure S2. A photomicrograph of a section of cerebral cortex of sham group showing delicate layer of pia matter (arrow), outer grey matter six layers (GM) of cerebral cortex
