# Supplementary material for: Novel Intranasal Drug Delivery: Geraniol Charged Polymeric Mixed Micelles for Targeting Cerebral Insult as a Result of Ischaemia/Reperfusion
Source: Pharmaceutics. 2020 Jan 17;12(1):76. doi: 10.3390/pharmaceutics12010076 (PMC7022886; doi:10.3390/pharmaceutics12010076)
Supplement: Supplementary file 1 [file pharmaceutics-12-00076-s001.zip › Figure S3.pdf]

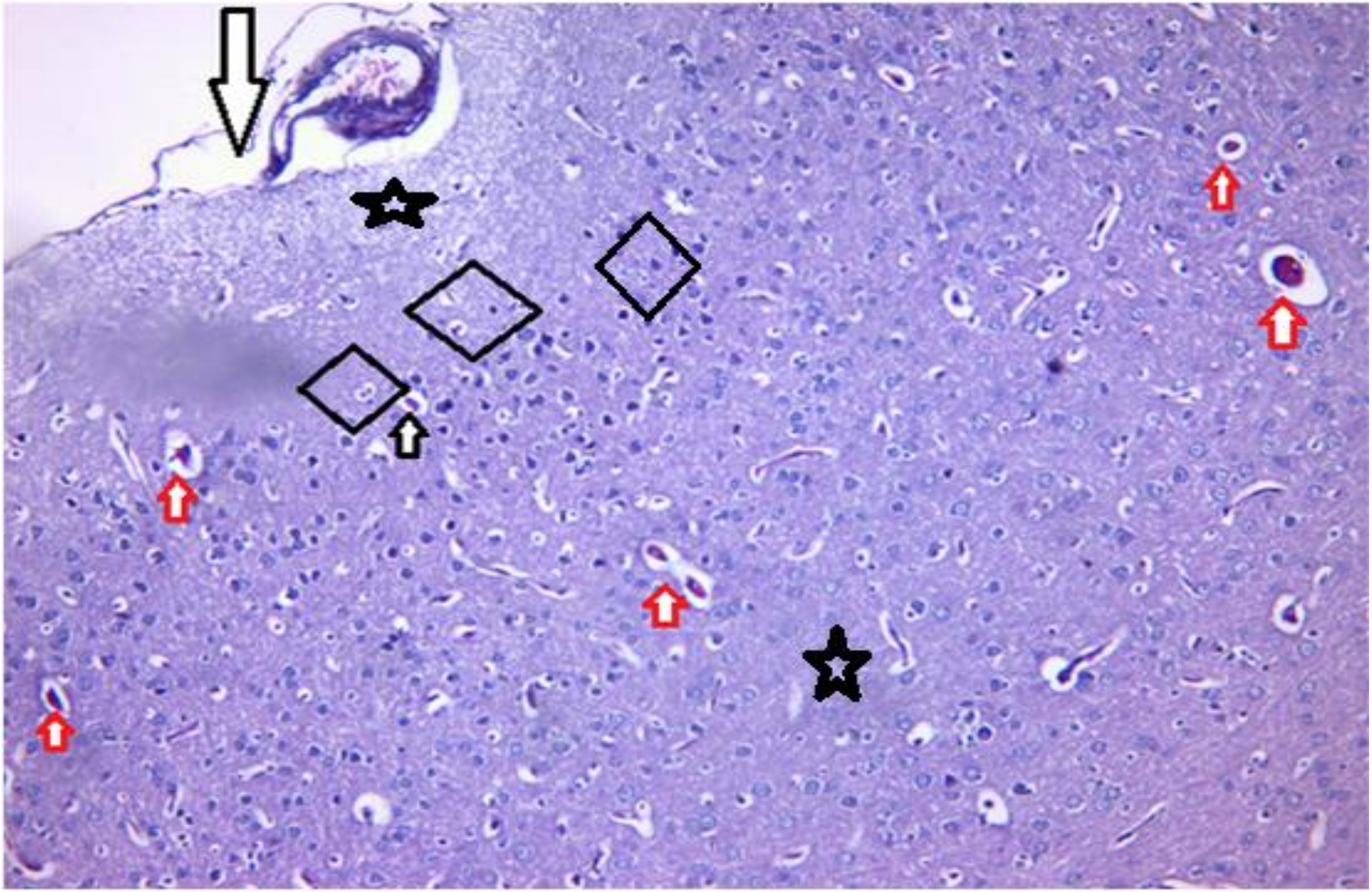

**Figure S3.** Photomicrograph of prophylactic vehicle group showed thickened and vascular pia matter (arrow), oedma under pia (star), red neuron infarction (red arrow) and many apoptotic cells
