# Supplementary material for: Novel Intranasal Drug Delivery: Geraniol Charged Polymeric Mixed Micelles for Targeting Cerebral Insult as a Result of Ischaemia/Reperfusion
Source: Pharmaceutics. 2020 Jan 17;12(1):76. doi: 10.3390/pharmaceutics12010076 (PMC7022886; doi:10.3390/pharmaceutics12010076)
Supplement: Supplementary file 1 [file pharmaceutics-12-00076-s001.zip › Figure S4.pdf]

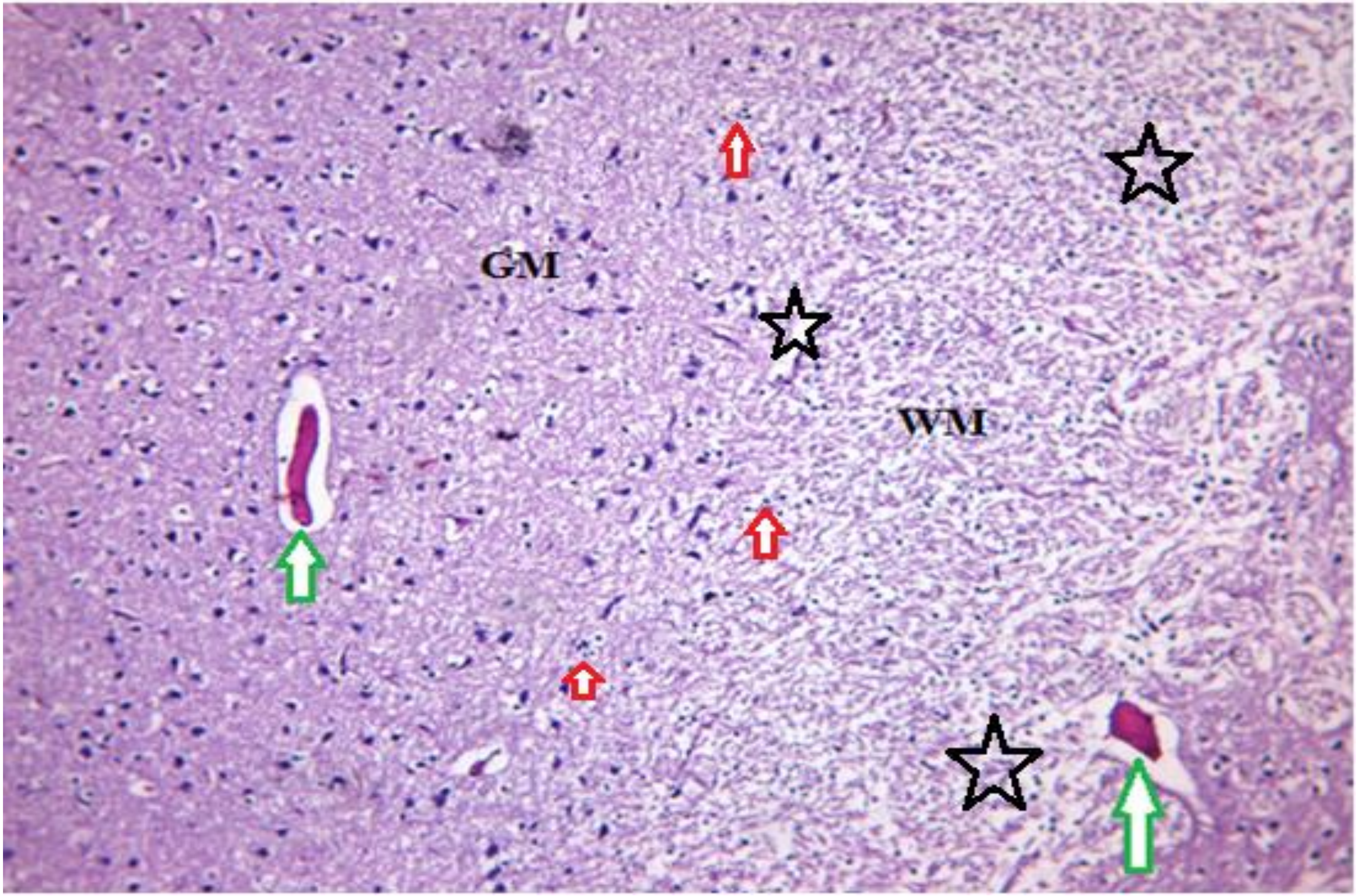

Figure S4. Photomicrograph of prophylactic Geraniol oil group showed infarction (green arrow) in grey matter (GM) and white matter (WM), many apoptotic and cellular infiltration (red arrow), oedema (stars)
