# Supplementary material for: Novel Intranasal Drug Delivery: Geraniol Charged Polymeric Mixed Micelles for Targeting Cerebral Insult as a Result of Ischaemia/Reperfusion
Source: Pharmaceutics. 2020 Jan 17;12(1):76. doi: 10.3390/pharmaceutics12010076 (PMC7022886; doi:10.3390/pharmaceutics12010076)
Supplement: Supplementary file 1 [file pharmaceutics-12-00076-s001.zip › Figure S5.pdf]

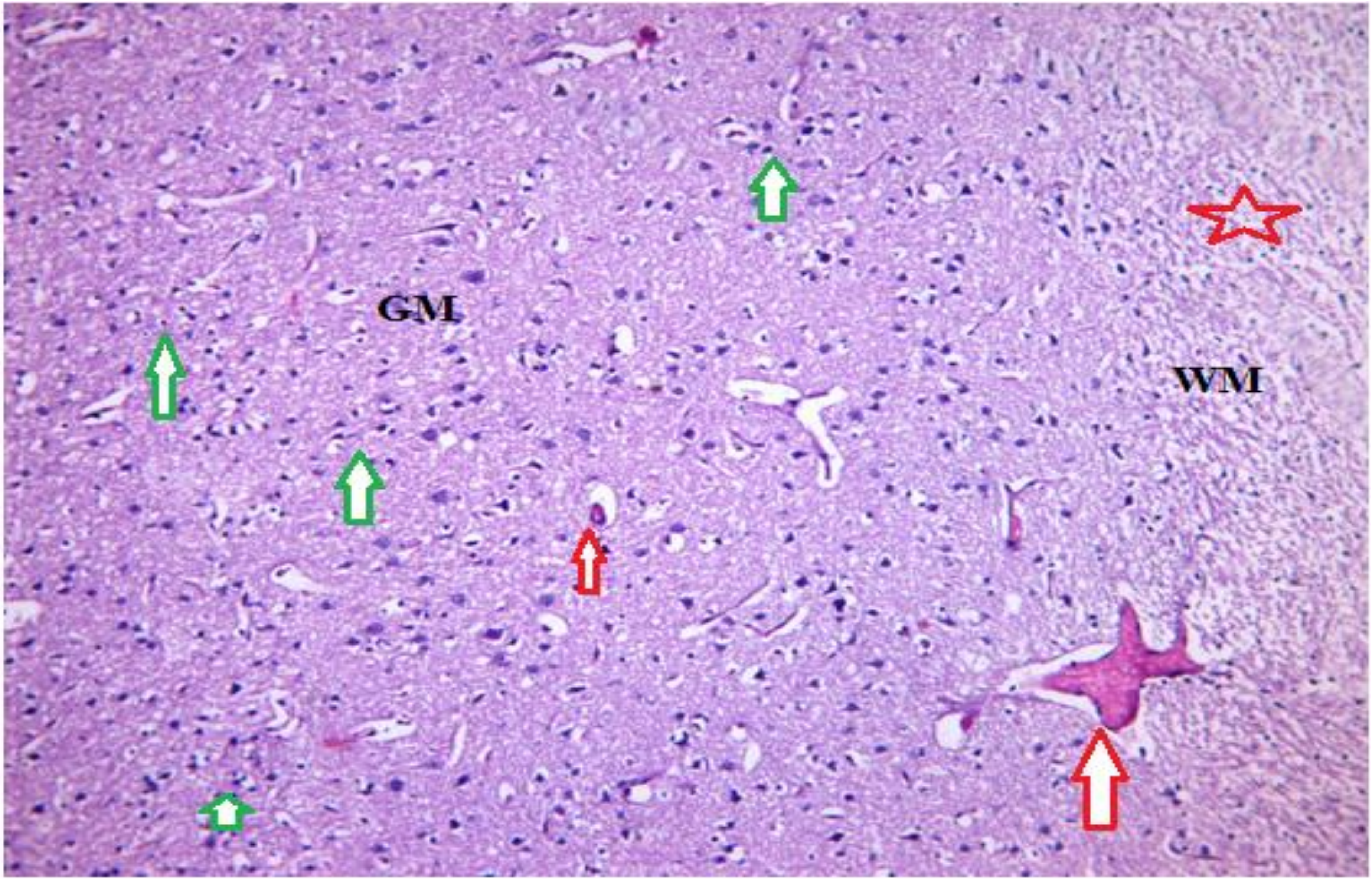

Figure S5. Photomicrograph of prophylactic Geraniol micelle (0.25ml) group shows infarction in small areas (red arrow), mild edema (star) mainly in white matter (WM) and increased cellular infiltration (green arrows)
