# Supplementary material for: Novel Intranasal Drug Delivery: Geraniol Charged Polymeric Mixed Micelles for Targeting Cerebral Insult as a Result of Ischaemia/Reperfusion
Source: Pharmaceutics. 2020 Jan 17;12(1):76. doi: 10.3390/pharmaceutics12010076 (PMC7022886; doi:10.3390/pharmaceutics12010076)
Supplement: Supplementary file 1 [file pharmaceutics-12-00076-s001.zip › Figure S6.pdf]

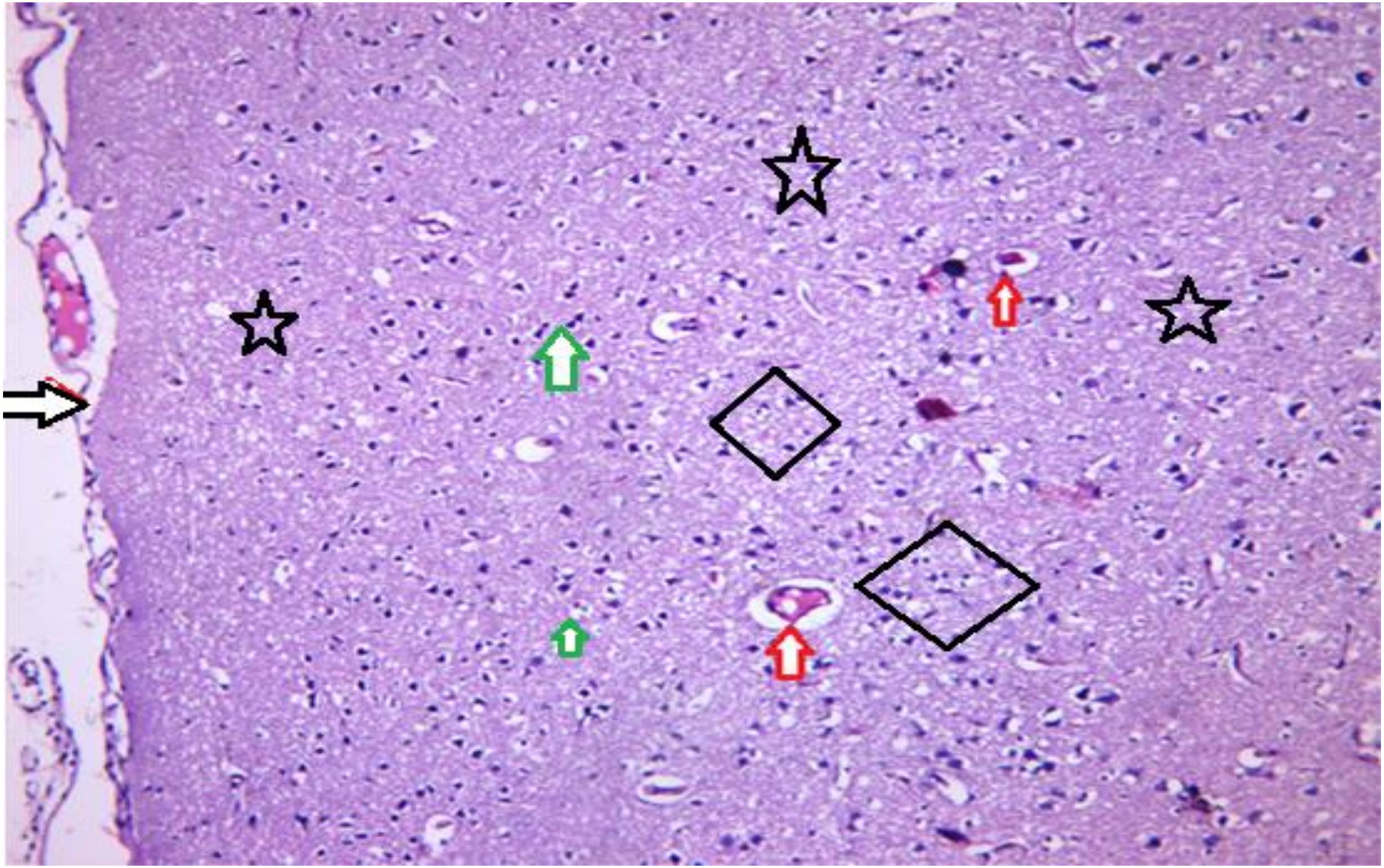

Figure S6. Photomicrograph of prophylactic Geraniol micelle (0.5ml) group showing pia matter with cellular infiltration, oedema (stars) red neuronal infarction (red arrow), cellular infiltration (rectangle), apoptotic and mitotic cells (green arrow)
