# Supplementary material for: Novel Intranasal Drug Delivery: Geraniol Charged Polymeric Mixed Micelles for Targeting Cerebral Insult as a Result of Ischaemia/Reperfusion
Source: Pharmaceutics. 2020 Jan 17;12(1):76. doi: 10.3390/pharmaceutics12010076 (PMC7022886; doi:10.3390/pharmaceutics12010076)
Supplement: Supplementary file 1 [file pharmaceutics-12-00076-s001.zip › Figure S7.pdf]

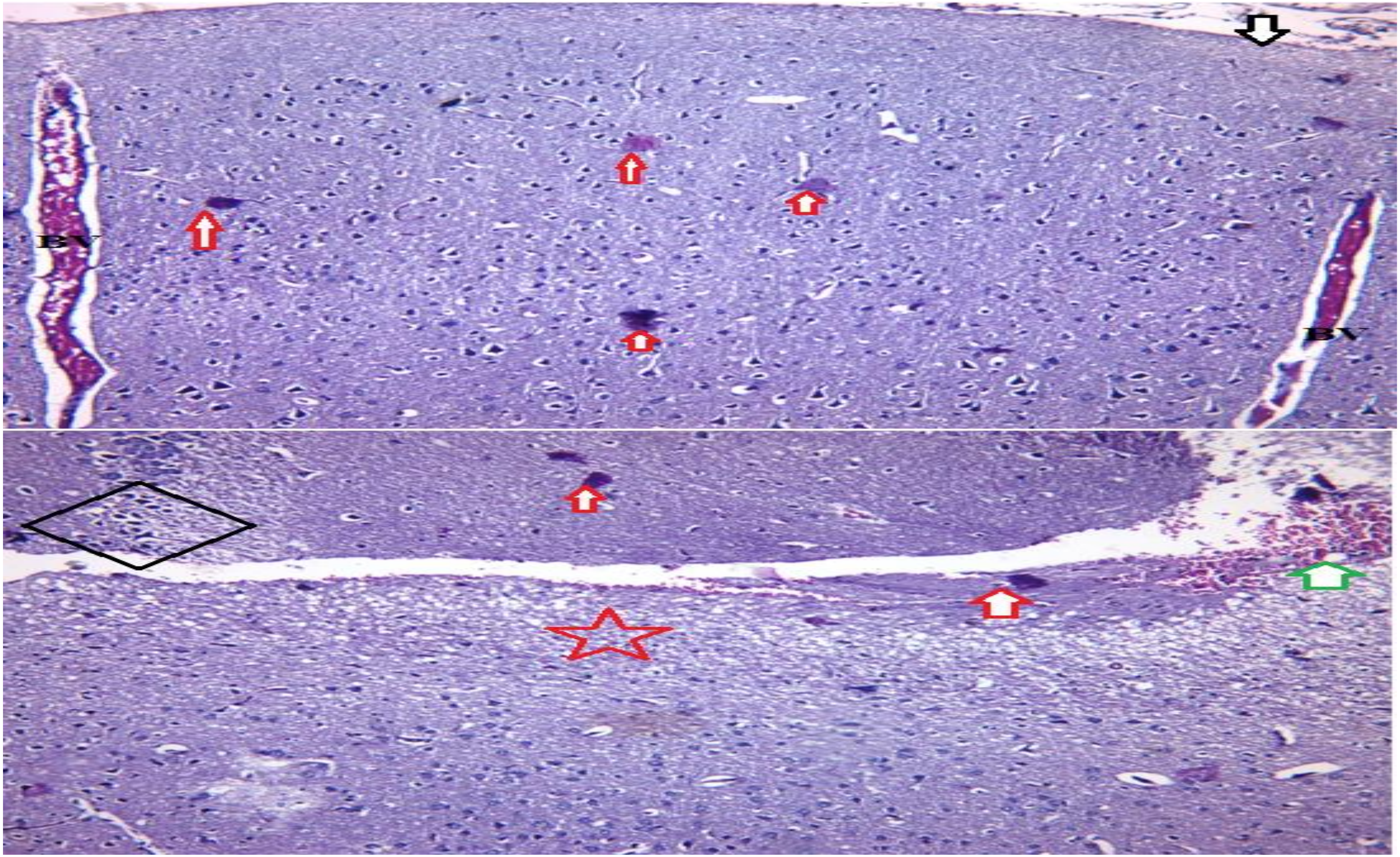

Figure S7. Photomicrograph of therapeutic Geraniol oil (0.02ml) showing thin pia (black arrow), many infarct areas (red arrows), high vascularity with congested blood vessels (BV) and haemorrhage (green arrow), oedema (star) note apoptotic cells (shape)
